# The swallowing reflex and its significance as an airway defensive reflex

**DOI:** 10.3389/fphys.2012.00489

**Published:** 2013-01-07

**Authors:** Takashi Nishino

**Affiliations:** Kaken HospitalIchikawa, Japan

**Keywords:** swallowing, pulmonary aspiration, upper airway, defensive reflexes, respiration

## Abstract

Swallowing function, in humans, is very complex. Swallowing plays, not only an important role in food digestion, but also a major role in preventing the entrance of food and/or other materials into the lower respiratory tract. To achieve this, precise coordination is necessary between breathing and swallowing since the pharynx serves as a common pathway for both respiration and digestion. The swallowing reflex consists of afferent pathways, central integration, and efferent pathways. Any defect or disorder along reflex arc can cause a potential delay or impairment in swallow function. The swallowing reflex can be modulated not only by pathological factors but also by physiological factors. Among these, timing of swallows in relation to the phase of respiration may be the most important factor that determines the occurrence of pulmonary aspiration, since phases of inspiration and the expiration-inspiration transition are the most vulnerable for pulmonary aspiration.

## Introduction

Respiration and swallowing utilize a common passageway and the two activities must be coordinated so that mutual compromise does not occur. A high degree of coordination between respiration and swallowing is required to maintain adequate ventilation without causing pulmonary aspiration. Like cough reflex, swallowing reflex has an obvious protective value against the aspiration of foreign materials into the respiratory tract (Nishino, 1993). The purpose of this paper is to discuss physiological and clinical factors that influence the swallowing reflex as an airway defensive reflex.

## Neural organization of the swallowing

The reflex control system of swallowing consists of afferent, central, and efferent components, and the integrity of the reflex control system seems to contribute to the prevention of pulmonary aspiration.

### Afferent pathways

The receptive regions for reflex swallowing include many locations in oro-pharyngeal space such as the soft palate, uvula, dorsa surface of the tongue, pharyngeal surface of the epiglottis, faucial pillars, glossoepiglottidinal sinus, dorsal pharyngeal wall, and the pharyngoesophgeal junction (Pommerenke, 1928; Storey, 1968; Sinclair, 1971; Mannson and Sandberg, 1974; Miller, 1982). Among these regions, the faucial pillars are the most sensitive region in humans. Receptors responsible for elicitation of swallowing reflex have not been identified histologically. However, specific fluid or water receptors and some slowly adapting pressure receptors are known to be distributed unevenly over the pharyngeal and laryngeal regions (Mathew and Sant'Ambrogio, 1988). It is likely that these receptors can initiate swallowing while responding to water and light touch. Primary afferents from the receptors in the oropharyngeal mucosa travel in the trigeminal (V), glossopharyngeal (IX), and vagus nerves (X), and converge in the solitary tract destined for synaptic contact with second-order neurons in the nucleus tractus solitarius (NTS). The swallowing reflex elicited by primary afferents may be modified by lung/chest wall receptors such as vagal and intercostals muscle afferents.

### Central integration

The NTS is not only an afferent portal but has interneurons that perform a more complex level of swallowing control. Although data has shown that only stimulation of the NTS and the adjacent reticular formation could elicit swallowing (Miller, 1972), there has been no evidence to suggest that the swallowing center resides within the NTS. Nevertheless, extensive evidence supports the existence of a swallowing center within the brain stem (Doty et al., 1967).

It is well established that the swallowing center receives descending influences from the cerebral cortex and subcortical areas. Descending fibers from the frontal cortex have been found histologically to terminate in widespread areas in the reticular formation of the brainstem (Kuypers, 1958). There is no doubt that the cerebral cortex and subcortex areas and their interaction with brain stem can play an important role in the neural regulation of swallowing reflex. The central control of swallowing is modified by peripheral feedback from the pharyngeal, laryngeal, and esophageal regions. Indeed, it has been demonstrated that pharyngeal stimulation can induce remarkable increases in the excitability of swallowing motor cortex (Sumi, 1969). The peripheral feedback may contribute to the maintenance of a general facilitative level of the swallowing center. Although the concept of the swallowing center is still valid, recent advances in neuroanatomic, neurochemical, and pharmacologic methodology suggest a model of circuitry representing a structurally defined central swallowing pattern generator (SPG) (Paton et al., 1999). The term SPG reflects the major conceptual reorientation toward the intrinsic operations for the central neurocircuitry associated with swallowing as an autonomous network. The premotor neurons that constitute the swallowing central pattern generator are highly interconnected to multiple areas of the brain stem and the central nervous system while providing a potential anatomic substrate integration of swallowing activity with airway protective reflexes (Broussard and Atschuler, 2000).

### Efferent pathways

The motor nuclei involved in swallowing are the trigeminal (V), facial (VII), ambiguous, and hypoglossal nuclei (XII). However, only a relatively small portion of trigeminal and facial nuclei participate in normal swallowing. In contrast, both ambiguous and hypoglossal nuclei activate all of their motoneurons during the act of swallowing and are considered to be the most important motor nuclei involved in this function (Miller, 1982). Inspiratory effort produced by a characteristic brief burst of phrenic nerve known as “Schluckatmung” is said to occur consistently in human infants (Wilson et al., 1981) and inconsistently in adult humans (Vantrappen and Hellemans, 1967). Thus, the phrenic nerve may also be involved in swallowing act (Nishino et al., 1985a).

## Factors affecting the swallowing reflex

### Muscle weakness

Swallowing causes reflex closure of the glottis, elevation of the larynx, and a transient cessation of respiration. These complex motor acts implicate that numerous muscles are activated during the act of swallowing. Muscle disorders such the myopathies, polymyositis, progressive muscular dystrophy, and neuromuscular junction disorders including a myasthenia gravis and myasthenic syndrome are known to lead to dysphagia when the muscles of swallowing are involved (Nishino, 1997). Severe oropharyngeal weakness and ineffective cough predisposes the patient with dysphagia to aspiration. Partial paralysis produced by muscle relaxants is a common clinical situation during perioperative periods. The results of human experiment demonstrated that compared with a non-paralyzed condition, there were remarkable decreases in mesopharyngeal pressure and submental EMG activity during partial paralysis, indicating that partial paralysis does impact swallowing function (Isono et al., 1991). However, general characteristic of swallowing pattern were essentially similar in both non-paralyzed and partially paralyzed conditions. For example, the latency, and the periods from the onset of the EMG change to the peak of pharyngeal pressure were similar, suggesting that partial paralysis does not influence the neural pathway of the swallowing reflex.

### Body position

**Figure 1:**
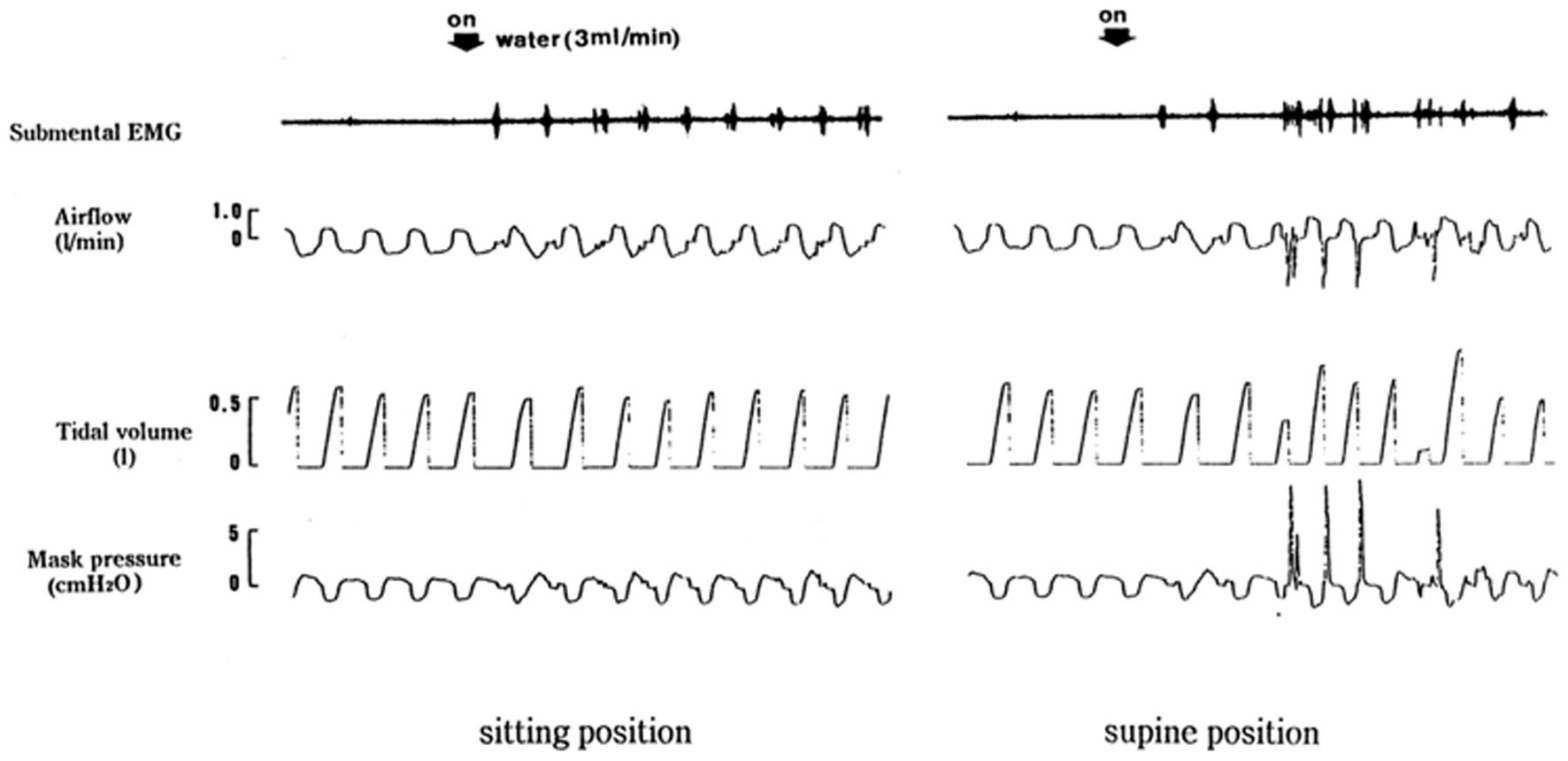
**Different swallowing responses in different body positions.** Respiratory and swallowing responses to continuous infusion of water into the pharynx were examined in an epiglottis-resected patient in both supine and sitting positions.

The epiglottis itself is not considered to be essential to effective swallowing function. In the absence of the epiglottis or normal epiglottic inversion, the hypolaryngeal complex is able to close the glottis and effectively protect the airway. Figure 1 shows different responses of swallowing to continuous infusion of water into the pharynx in a patient who underwent the resection of the epiglottis due to carcinoma of the epiglottis. We examined the effects of posture on swallowing response to continuous infusion of water in this patient. In a sitting position, a normal swallowing response to water instillation was observed in this patient. However, in a supine position this patient showed a vigorous coughing immediately after the start of water infusion, possibly indicative of discoordinated laryngeal closure and subsequent penetration or aspiration to the airway. Detailed analysis of the timing of swallows during continuous infusion of water shows that all the swallows in the sitting position took place at the expiration phase. On the other hand, in the supine position, the swallow coincided with inspiration initiated cough reflex. These findings suggest that in patients without the epiglottis, body position may affect the timing of swallows which plays an important role in protection of the airway from aspiration of swallowed material. Although human physiology may need gravity for pharyngeal movement of the bolus, it has been reported that in normal healthy subjects gravity does not influence pharyngeal bolus transport (Johnsson et al., 1995).

### Timing of swallow

In order to analyze the timing of swallows, types of swallows were classified into four different types according to swallow-associated airflow pattern. I swallow is inspiratory swallow occurring during inspiratory phase. I-E swallow is the swallow occurring during inspiratory-expiratory transition phase, E swallow is expiratory swallow occurring during expiration, and E-I swallow is the swallow occurring during expiratory-inspiratory transition phase (Kijima et al., 1999; Nishino et al., 1999; Sai et al., 2004).

In normocapnic awake subjects the majority of swallows interrupt respiration in the expiratory phase (Nishino et al., 1985b, 1999; Sai et al., 2004) whereas in patients breathing spontaneously under general anesthesia with sevoflurane, tendency to time swallows during expiration is lost (unpublished observation), suggesting that the consciousness is an important factor for causing the preferred timing of swallows during expiratory phase. The loss of the preponderant coupling of swallows with the expiratory phase under unconscious state may explain in part the predisposition to aspiration during unconsciousness.

We have shown previously that during continuous infusion of water into the pharynx, the frequency of swallowing decreases and the incidence of laryngeal irritations increases with increasing levels of P_ET_co_2_. Hypercapnia not only decreases the swallowing rate but also alters the timing of swallows in relation to the phase of the respiratory cycle. Analysis of the timing of swallows in relation to the phase of the respiratory cycle during continuous infusion of water showed that during normocapnia the majority of swallows occurred at expiratory and I-E transition periods whereas increases in Pco_2_ considerably modified the types of swallows (Nishino et al., 1999). Thus, E swallows progressively decreased with a progressive increase in ventilation and Pco_2_, whereas I and E-I transition swallows progressively increased with increasing level of Pco_2_. Moreover, as the Pco_2_ level increased so did the incidence of laryngeal irritation. The majority of laryngeal irritations occurred either when the swallows were initiated at E-I transition or when the swallows coincided with inspiration. These findings suggest that the timing of swallows with respiration may be an important determinant of effective airway protection. In other words, I swallows and E-I swallows are the most dangerous swallows for aspiration of swallowed materials (Paydarfar et al., 1995; Nishino et al., 1999).

### Changes in respiratory mechanics

Changes in respiratory mechanics and an addition of respiratory loads to the respiratory system are frequently observed in several clinical situations. It has been shown that an addition of respiratory load changes breathing patterns, and the breathing patterns differ markedly depending on the type of respiratory loading. For example, breathing patterns during flow-resistive loading are characterized by slow, deep breathing, whereas those during elastic loading are characterized by rapid shallow breathing (Mead, 1960). When the respiratory pattern and reflex swallowing elicited by continuous infusion of water were analyzed under different conditions, i.e., the control condition without respiratory loading, with resistive loading, and with elastic loading, it was demonstrated clearly that flow-resistive loading causes a decrease in respiratory frequency and a decrease in swallowing rate, whereas elastic loading causes a rapid, shallow breathing with an increase in swallowing rate (Kijima et al., 1999). Furthermore, when the distribution of the timing of swallows in reference to the phase of the respiratory cycle for each respiratory load were analyzed, half of the swallows occurred at the I-E transition during flow-resistive loading and at the E-I transition during elastic loading, whereas swallows during expiratory phase were most commonly observed under the control condition. In addition, the incidence of laryngeal irritation during elastic loading was much higher than that during control condition or resistive loading. This finding is again in agreement with the finding of Paydarfar et al. (1995) who showed E-I swallows are the most dangerous swallows for aspiration of swallowed materials.

### Changes in lung volume

In order to see modulation of swallowing reflex by lung volume changes, we studied the effects of changes in lung volume produced by several maneuvers like application of extrathoracic negative pressure (Kijima et al., 2000), voluntary hyperventilation, breath-holding, and airway occlusion at FRC (Yamamoto and Nishino, 2002; Sai et al., 2004). The result of our experiments showed that both application of extrathoracic negative pressure and voluntary hyperpnea cause a marked decrease in swallowing rate during repetitive swallowing. In contrast, withdrawal of phasic vagal influence either by breath-holding or airway occlusion causes a considerable increase in swallowing rate during repetitive swallowing. These findings suggest that the attenuation of the swallowing reflex during the increased lung volume is probably produced by a lung-volume-related, vagally-mediated reflex effect. The results of animal experiments using new born lambs generally support the notion that the swallowing reflex is inhibited by stimulation of vagal receptors (Samson et al., 2008).

## Clinical implications of the swallowing reflex

A variety of etiological insults to any part of the reflex arch consisting of afferent pathways, central integration, and efferent pathways of the swallowing reflex cause impairment of triggering reflex swallowing. It has been shown that the frequency of swallowing decreases during sleep (Lear et al., 1965). Also, it has been demonstrate that aspiration of pharyngeal secretions easily occurs in normal adults during deep sleep (Huxley et al., 1978), presumably due to depression of pharyngeal reflexes, including the swallowing reflex. A light level of sedation causes prolongation of the latency for initiation of the swallowing reflex and a decrease in the number of swallows elicited, indicating depression of the swallowing reflex (Nishino et al., 1987). Similar changes in swallowing reflex were observed even in animal experiment (Nishino et al., 1985a), indicating that the impairment of the swallowing reflex can be evaluated by measuring the latency and number of swallows elicited in response to swallowing stimulus.

It has been likewise shown that patients with obstructive apnea syndrome exhibit an impaired swallowing reflex probably due to the perturbed neural and muscular function of the upper airways (Teramoto et al., 1999).

Impairment of the swallowing reflex may occur following surgical operations on the head and neck due to resection of critical structures involved in the swallowing process or sacrifice of afferent and/or efferent neural pathways (Conley, 1960; Levine, 1988).

Impairment of the swallowing reflex can also occur without a depression of central nervous system or without an apparent neurologic damage. Application of nasal CPAP has been shown to cause a great decrease in the frequency of swallows (Nishino et al., 1989). It has been also reported that a nasal CPAP inhibits non-nutritive swallowing in newborn lambs during quiet sleep (Samson et al., 2005). These findings may have some important clinical implications. For example, nasal CPAP is administered frequently in the recovery room or in the intensive care unit, and any predisposition to aspiration by CPAP due to depression of the swallowing reflex is particularly unfavorable in these clinical settings.

Patients with Parkinson's disease show a prolonged triggering of the swallowing reflex (Potulska et al., 2003), suggesting that problems exist within the central nervous system including the extrapyramidal system. However, these patients also show a prolonged duration of the pharyngeal reflex time due to slowness of the sequential muscle movement, particularly those of suprahyoid-submental muscles while cricopharyngeal muscles of the upper esophageal sphincter is found to be electrophysiologically normal (Ertekin et al., 2002). It is possible that the swallowing problems of Parkinson's disease are related not only with abnormalities in the central pattern generator but also with dysfunction of basal ganglia, dysfunction of efferent pathways, and abnormalities in swallowing muscles.

## Control system of the swallowing reflex in humans

**Figure 2:**
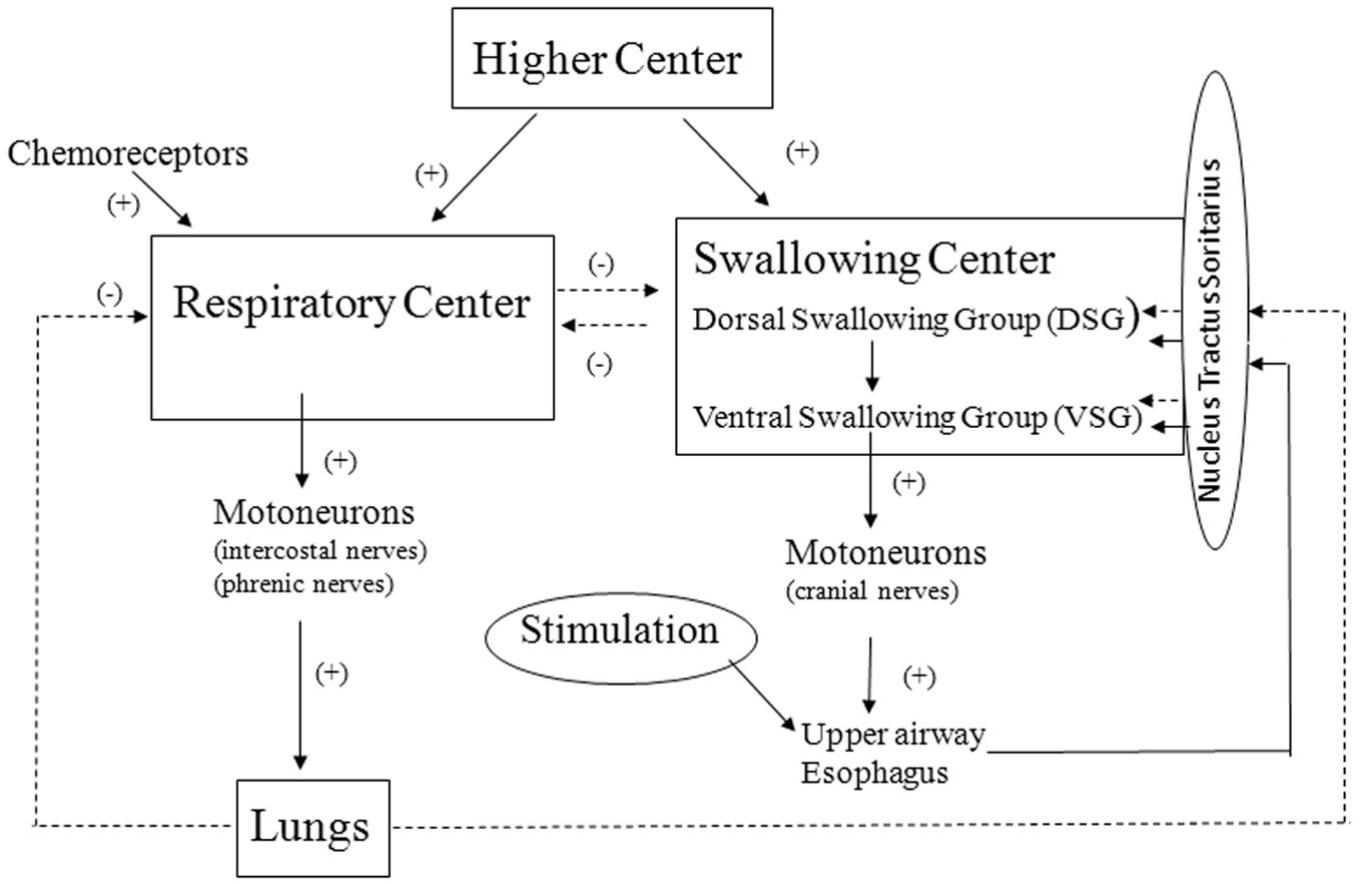
**Framework of the control of reflex swallowing.** Solid line and broken line represent facilitative and inhibitory pathways, respectively.

Based on the results obtained in human experiments, a conceptual framework of the control of reflex swallowing was constructed (Figure 2).

First, stimulation of upper airway mucosa activates chemical or mechanical receptors in the upper airway (Mathew and Sant'Ambrogio, 1988). Afferent impulses from the peripheral receptors converge into the NTS and then into the dorsal swallowing neurons (Broussard and Atschuler, 2000; Jean, 2001). The swallowing neurons in the dorsal group are likely involved in the initiation and the programming of swallowing. The swallowing neurons of the ventral group receive the swallowing input from the dorsal neurons, and these neurons are probably switching neurons that distribute the swallowing excitation to the various pools of motoneurons involved in swallowing (Jean, 2001).

Second, the swallowing neurons in the dorsal group also receive the central inputs from the higher centers (Kuypers, 1958; Miller, 1982). The finding that the preponderant coupling of swallows with expiration is lost in anesthetized condition suggests that the higher centers contribute to the determination of timing swallows.

Third, there is an interaction between the respiratory center and the swallowing center so that each center exerts reciprocally an inhibitory influence (Miller, 1982). This interaction may also contribute to the determination of timing of swallows together with the influence of the higher centers controlling the behavioral responses.

Finally, changes in lung volume modulate the control of swallowing rate as well as the timing of swallowing in reference to the respiratory cycle (Kijima et al., 2000) and this is probably vagal reflex effect. It is well known that the Hering–Breuer inflation reflex is very weak in man and does not play an important role in the control of respiration in normal physiological condition (Guz et al., 1966). In contrast, the lung volume-related reflex control of swallowing seems to be operative in normal physiological condition in man.

## Concluding remarks

- Swallowing reflex serves as a defensive airway reflex.
- Any procedures that disturb the coordination of respiration and swallowing may increase the chance of pulmonary aspiration.
- Timing of swallows in relation to the phase of respiration may be an important factor that determines the occurrence of pulmonary aspiration, and phases of inspiration and expiration-inspiration transition are the most vulnerable for pulmonary aspiration of swallowed materials.
- Lung volume-related vagal reflex plays an important role in the control of reflex swallowing.

### Conflict of interest statement

The author declares that the research was conducted in the absence of any commercial or financial relationships that could be construed as a potential conflict of interest.
